# Incidence of PEG-Asparaginase-Induced Pancreatitis in Children During Acute Lymphoblastic Leukemia Treatment: A Multicenter Study

**DOI:** 10.7759/cureus.89198

**Published:** 2025-08-01

**Authors:** Hanan M Alrasheedi, Farah Malaeb, Sameh Awwad, Ali Alnakhli, Nourah Alrashidi, Rawand Alharbi, Dema Almolaiki, Zainab S Alahmari, Ghadah M Alyousif, Hasan Alshakhs, Renad A ALfouzan, Abdulrahman K Alajlan, Fatima Alhaddad, Nora Alqussair, Saad Daama, Nasseem Al Ali, Abeer Al Amer, Murtada H Alsultan

**Affiliations:** 1 Pediatric Hematology and Oncology, King Fahad Medical City, Riyadh, SAU; 2 Clinical Pharmacy, King Fahad Medical City, Riyadh, SAU; 3 Pharmaceutical Care, King Abdulaziz Medical City - Riyadh, Riyadh, SAU; 4 Pediatric Hematology and Oncology, King Fahad Specialist Hospital, Dammam, SAU; 5 Clinical Pharmacy, King Abdulaziz Medical City - Riyadh, Riyadh, SAU; 6 Pharmaceutical Care, King Fahad Medical City, Riyadh, SAU; 7 Pediatrics, King Abdulaziz Medical City - Riyadh, Riyadh, SAU; 8 Pharmaceutical Care, King Fahad Specialist Hospital, Dammam, SAU

**Keywords:** all, asparaginase, pancreatitis, pediatric, treatment

## Abstract

Background: Acute lymphoblastic leukemia (ALL) is the most common pediatric cancer globally. PEG-asparaginase (PEG-ASPA), a cornerstone of ALL treatment, significantly improves outcomes but is associated with serious toxicities, including acute pancreatitis (AP). This study evaluates the incidence and associated risk factors of PEG-ASPA-induced AP among pediatric ALL patients in Saudi Arabia.

Methods: This multicenter retrospective cohort study included pediatric ALL patients treated with PEG-ASPA between January 2019 and October 2023 at three tertiary Saudi centers. Data included demographics, risk stratification, PEG-ASPA doses, and toxicity. AP was defined per CTCAE v5.0 criteria. A multivariate logistic regression model was used to evaluate risk factors.

Results: Of 322 pediatric patients, 5% (n=16) developed AP. Events were more frequent in children >10 years old (44%) and during consolidation (38%) and interim maintenance 2 (38%) phases. Multivariate analysis showed that age >10 years (OR=3.65), high-risk protocols (OR=2.07), and >5 PEG-ASPA doses (OR=1.58) were associated with increased AP risk. Most cases were grade 2 or 3 and resolved with supportive care.

Conclusion: The incidence of PEG-ASPA-induced AP among pediatric ALL patients in Saudi Arabia is consistent with international data. Higher age, intensive protocols, and cumulative dosing may increase risk. Routine monitoring of pancreatic enzymes and triglycerides prior to PEG-ASPA administration is recommended to enhance early detection and reduce the risk of severe toxicity.

## Introduction

Acute lymphoblastic leukemia (ALL) is the most common childhood cancer, and survival rates have significantly improved with risk-adapted chemotherapy protocols that include asparaginase as a key component [1].

Asparaginase plays a critical role by depleting serum asparagine, an amino acid essential for leukemic cell survival. However, it is sometimes associated with significant adverse effects, namely thrombosis and acute pancreatitis, which can result in therapy delays, modifications, or permanent discontinuation, ultimately impacting treatment outcomes [2,3].

Asparaginase-induced acute pancreatitis (AP) is a well-recognized but unpredictable complication. The pathophysiology is not fully understood, but proposed mechanisms include direct cytotoxic effects on pancreatic acinar cells, immune-mediated injury, and hypersensitivity reactions [4]. The incidence of AP varies across studies, ranging from 2% to 18%, and risk factors include older age, obesity, and certain genetic polymorphisms [5]. Current literature on AP predominantly stems from Western populations, with limited data from other ethnic or geographic cohorts [6].

In Saudi Arabia and the broader Middle East, epidemiological data on AP remain scarce, and no multicenter studies have been published to quantify its burden or evaluate its clinical course. Given differences in population genetics, healthcare delivery, and treatment protocols, findings from other regions may not be fully generalizable. Furthermore, understanding the incidence and consequences of AP in our local context is essential for forming evidence-based supportive care strategies and optimizing leukemia outcomes.

This study addresses this critical knowledge gap by providing multicenter data on the incidence, clinical presentation, and outcomes of AP in Saudi pediatric ALL patients. It also allows for comparison with international findings, enhancing the global understanding of this serious toxicity.

## Materials and methods

This observational retrospective study was conducted at three major healthcare institutions in Saudi Arabia: King Fahad Medical City (KFMC) Comprehensive Cancer Center in Riyadh, King Abdullah Specialist Children Hospital (KASCH) in Riyadh, and King Fahad Specialist Hospital (KFSH) in Dammam. Thorough discussions prior to initiating the data collection were carried out, and thus the disagreements were resolved. Ethical approval was obtained from institutional review boards for all participating hospitals. We utilized the hospital's electronic health record system to conduct a comprehensive chart review to include all pediatric patients aged 1 to 14 years diagnosed with either T-cell or B-cell ALL who received PEG-asparaginase (PEG-ASPA) therapy between January 2019 and October 2023.

Exclusion criteria included patients with infantile ALL (aged <1 year), those with Down syndrome, individuals older than 15 years at diagnosis, and those for whom asparaginase was not included in their treatment regimen. The grading of PEG-ASPA-related toxicities was performed in accordance with the National Cancer Institute Common Terminology Criteria for Adverse Events (CTCAE), version 5.0, and this grading was validated by two independent reviewers to ensure accuracy and reliability.

Premedication protocols were adhered to as per the Children’s Oncology Group (COG) guidelines before each administration of PEG-ASPA to mitigate potential infusion-related reactions. At our three centers, a standardized premedication regimen was followed, which included corticosteroid and antihistamine.

Sample size calculations were performed to ensure sufficient power to detect an incidence rate of 5% for AP in pediatric patients treated with PEG-ASPA. Previous literature indicated an incidence range of AP between 2% and 10% [5], leading to an estimated sample size of 300 patients required to achieve 80% statistical power with a margin of error of 2%.

Ultimately, a total of 322 patients were included in the final analysis, enabling robust statistical evaluations regarding the incidence and associated toxicities of PEG-ASPA therapy.

Data analysis

Statistical analyses were conducted using IBM SPSS Statistics for Windows, Version 22 (Released 2013; IBM Corp., Armonk, New York, United States). Descriptive statistics were reported as frequencies (n) and percentages (%). Continuous variables were summarized as mean standard deviation (SD). The chi-square (chi-square) test was used to assess associations between categorical variables. One-way ANOVA was applied to compare mean values across groups. A p-value of <0.05 was considered statistically significant. All statistical analyses were two-tailed.

## Results

A total of 322 pediatric patients diagnosed with ALL were included in this study. The cohort comprised 60% male patients (n=192) and 40% female patients (n=130), indicating a higher prevalence of ALL in male patients. The age distribution of the patients was as follows: 39.9% (n=129) were aged between one and five years, 34.9% (n=113) were between six and nine years, and 24.7% (n=80) were older than 10 years (Table 1). B-cell ALL was the predominant subtype, accounting for 83% (n=266) of cases, while T-cell ALL comprised 17% (n=56). Patients were stratified into standard-risk (51%, n=165), high-risk (43%, n=140), and very high-risk (5%, n=17) groups based on their treatment protocols. Treatment protocols varied, with the AALL0331 protocol applied in 51% of cases (n=164), AALL0232 in 27% (n=89), AALL0434 in 17% (n=54), and AALL0932 in 5% (n=15).

**Table 1:** Demographic features of the study participants Data are presented as number (N) and percentage (%) or mean ± standard deviation (SD). Statistical comparisons were made using chi-square tests for categorical variables. A p-value <0.05 was considered statistically significant.

Characteristics	N (%)	p-Value	Test Statistic
Total number of patients	322		
Gender		0.016	chi-square = 5.76
Male	192 (60%)		
Female	130 (40%)		
Age group (in years)	(Mean ± SD) 6.8± 3.2 years	0.348	chi-square = 2.11
1-5	129 (39.9%)		
6-9	113 (34.9%)		
More than 10	80 (24.7%)		
Type of leukemia		0.040	chi-square = 4.23
T-cell leukemia	56 (17%)		
B-cell leukemia	266 (83%)		
Risk stratification		0.045	chi-square = 6.19
Standard risk	165 (51%)		
High risk	140 (43%)		
Very high risk	17 (5%)		
Treatment protocol used		0.649	chi-square = 1.64
AALL0331	164 (51%)		
AALL0434	54 (17%)		
AALL0232	89 (27%)		
AALL0932	15 (5%)		
Incidence of acute pancreatitis	16 (5%)		
CTCAE grading			
Grade 1	9 (56%)		
Grade 2	7 (44%)		
Grade 3	0		

**Figure 1:**
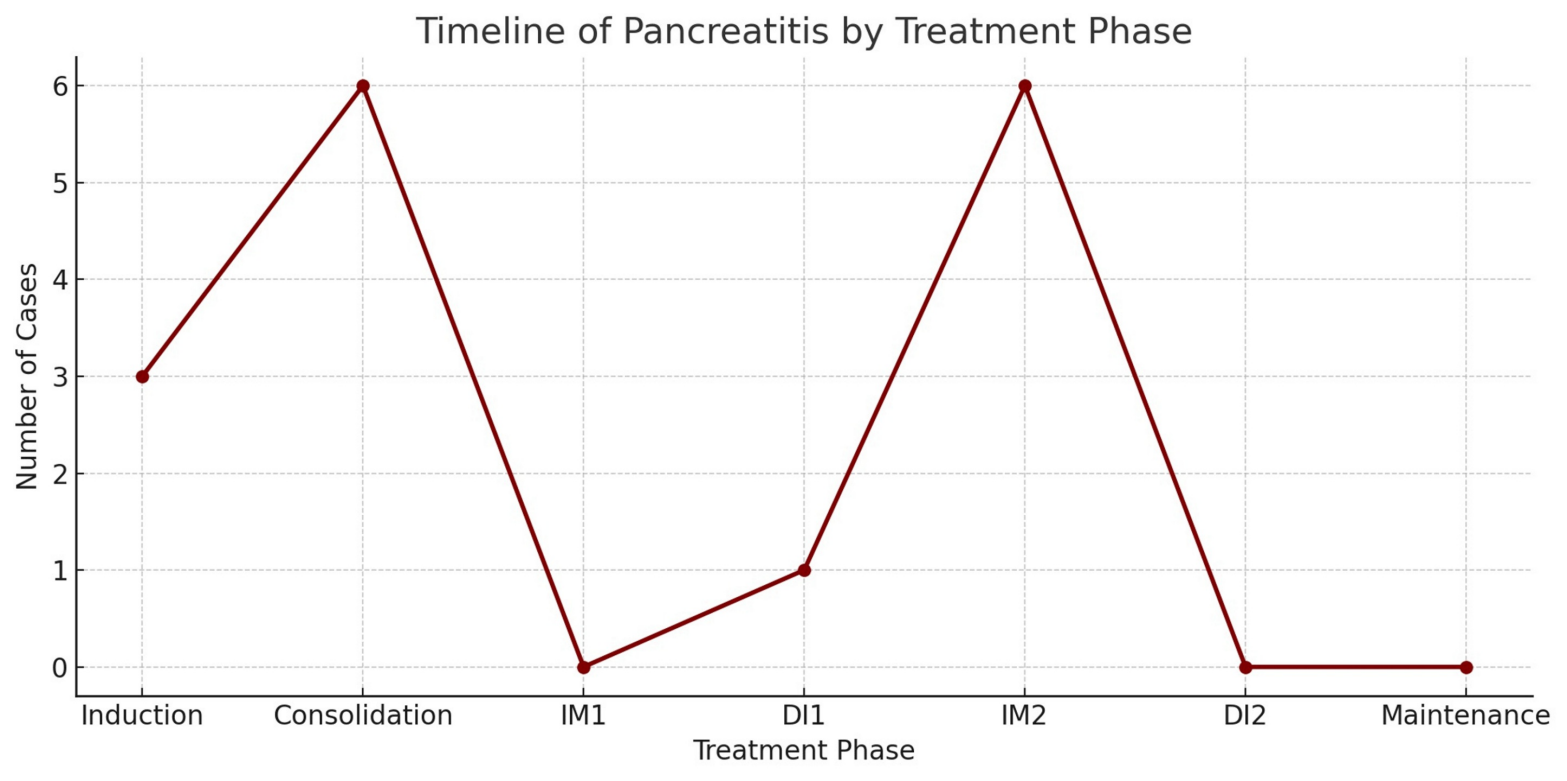
Timeline of pancreatitis by treatment phase IM: Interim maintenance

AP was identified in 5% (n=16) of patients, with 44% (n=7) of cases occurring in patients older than 10 years. AP was most commonly observed during the consolidation (38%, n=6) and interim maintenance 2 phases (38%, n=6), with fewer cases during the induction phase (19%, n=3) (Figure 1).

The severity of AP was graded using the CTCAE criteria, with 56% (n=9) of cases classified as Grade 2 and managed with supportive care, while 44% (n=7) were Grade 3, necessitating therapy modifications. Despite these occurrences, 97% (n=314) of patients maintained clinical stability throughout treatment, with only 3% (n=8) experiencing relapse or death due to other factors.

Laboratory findings revealed elevated amylase or lipase levels in 71% of AP cases, while imaging findings consistent with AP were reported in 68%. Regression analyses indicated no significant association between cumulative PEG-ASPA doses and the occurrence of AP.

The imaging results showed a 25% incidence of positive AP from CT findings and a 43% occurrence from ultrasound abdomen screenings.

Statistical analyses of these imaging methods yielded non-significant results, with p-values of 0.8 for CT and 0.2 for ultrasound.

All patients received the same dose of 2500IU/m^2^ of PEG-ASPA. Patients received a variable number of doses throughout their treatment; 76% of patients received 1-5 doses, 20% received 6-8 doses, and 6% received more than eight doses as shown in Table 2. Five percent of the patients developed toxicity from PEG-ASPA, mostly during the phases of induction, consolidation, and interim maintenance 2.

**Table 2:** Characteristics of PEG-asparaginase dosing Data are presented as number (N) and percentage (%). The timing of toxicity was recorded relative to treatment phase in patients diagnosed with pancreatitis (n=16). The majority occurred during the consolidation and interim maintenance 2 phases. No statistical significance was found between number of doses and symptom presentation (p > 0.05).

Characteristics	N (%)
Dose provided 2500IU\m^2^	322 (100%)
Number of doses received	
1-5 doses	245 (76%)
6-9 doses	66 (20%)
More than eight doses	11 (6%)
Time/phase of toxicity (n=16)	
Induction Phase	3 (18%)
Consolidation phase	6 (38%)
Interim maintenance 1 phase	0
Delayed intensification 1 phase	1(6%)
Interim maintenance 2 phase	6 (38%)
Delayed intensification 2 phase	0
Maintenance phase	0

ANOVA analysis showed no significant relationship between PEG-ASPA doses and symptoms of AP, with P-values of 0.8 for abdominal pain and 0.125 for back pain.

Toxicities were reported in 87% (n=14) of patients, mostly during consolidation and IM 2 phases (38% each), followed by induction (18%).

Symptoms included abdominal pain (87%, n=14), typically occurring within 1-3 days post-PEG-ASPA (38%, n=6) or after four weeks (19%, n=3). Back pain was reported in 25% (n=4) of patients.

Multivariate regression revealed independent risk from age >10 years (OR 3.65), high-risk therapy (OR 2.07), and more than five PEG-ASPA doses (OR 1.58) (Table 3).

**Table 3:** Multivariate logistic regression predicting pancreatitis

Variable	Coefficient	Odds Ratio
Intercept	-0.06	0.94
Age group over 10	1.29	3.65
High-risk protocol	0.73	2.07
Doses over 5	0.46	1.58
Male	0.57	1.77

Our study revealed a 5% incidence of AP, with abdominal pain being a common symptom. Upgrading therapy to high-risk or very high-risk categories was a significant risk factor for AP. Increased doses of PEG-ASPA were associated with higher AP incidence and abnormal liver enzymes (AST, ALT) but did not affect overall patient health. Most patients with AP received supportive and conservative care. According to CTACE grading, 56% (n=9) had grade 2 pancreatitis, and 44% (n=7) had grade 3. Switching 3% of patients (n=10) to Erwinia resulted in no toxicities. The overall stability was 97%, with only 3% experiencing relapse or death.

## Discussion

The 5% incidence of AP in children aged 1-14 years, diagnosed with ALL and treated with asparaginase-based regimens, aligns with international studies (2-10%) [5].

 Our study highlights that AP risk peaks during consolidation and IM2 phases, possibly due to cumulative dosing or immunosuppressive synergy.

The age distribution in our study highlights that a significant proportion of the patients were in the younger age brackets, particularly those under five years old (40%).

Treepongkaruna et al. identified high-risk chemotherapy as the only risk factor for developing AP [7]. Older age and intensified protocols were significant risk factors, consistent with prior findings by Pieters et al. and Chen et al [5,8]. Pieters et al. reported an overall AP incidence of 8.3% in pediatric ALL patients, of which 7.3% were asparaginase-related [8]. L-asparaginase-related pancreatitis typically emerged after a median of six doses, with a higher mortality rate reaching 44% in comparison to patients who did not experience AP [5,8].

Pharmaceutical-induced pancreatitis presents in approximately 5-20% of pediatric ALL cases. The mechanism remains unclear but may relate to asparagine depletion, disrupting pancreatic protein synthesis or increased triglycerides. This study is the first Saudi multicenter effort and benefits from robust data validation. However, retrospective design, lack of uniform imaging, and age cutoff (<15y) limit generalizability. Routine enzyme and triglyceride monitoring before PEG-ASPA, especially after dose 3, is recommended [8].

In our study, AP was diagnosed mainly during the consolidation and IM2 phases, each accounting for 38% of cases, followed by the induction phase at 18%, occurring after two to eight doses of PEG-asparaginase. This differs slightly from international data, where AP more commonly arises during early treatment phases. Denton et al. observed that complications were particularly prevalent during induction, affecting about 25% of patients [9]. Chen et al. detected AP in eight patients within 45 days of starting therapy [5]. Awwad et al., in a single-center study, found an AP incidence of approximately 3%, with most cases emerging during primary or secondary exposure to asparaginase [10]. Chen et al. also reported a positive correlation between AP incidence and the peak dose intensity of L-asparaginase, rather than cumulative dose [5]. Similarly, Wang et al. investigated recurrence patterns and associated risk factors in patients treated under SCCLG-ALL-2016 protocols. They emphasized age as a critical factor for AP risk and noted recurrence following the second exposure to PEG-asparaginase [11].

The main symptoms encountered by our patients included abdominal and back pain, consistent with global literature. Awwad et al. reported that abdominal pain, nausea, vomiting, hypocalcemia, hypoalbuminemia, and abnormal coagulation activity were predominant symptoms [10]. Abdominal pain is one of the earliest and most important indicators of pediatric pancreatitis. Our study findings aligned with these reports, with abdominal pain being the most common presenting symptom. Ziegler et al. similarly reported abdominal pain in 82% of pancreatitis cases [12].

In terms of toxicity, most AP cases in our cohort were grade 2 or 3 based on CTCAE grading, and some patients were switched from PEG-asparaginase to Erwinia. The overall clinical stability exceeded 90%, reflecting favorable treatment tolerability. We observed that modifications to high- and very-high-risk treatment protocols were associated with increased AP risk. Furthermore, our findings indicated a variable correlation between liver and pancreatic dysfunction, with 31- 50% of patients experiencing hepatotoxicity and 5% developing pancreatic toxicity. Awwad et al. recorded 79 toxicity episodes, with hypersensitivity accounting for 36.7%, hepatotoxicity for 31.6%, and both pancreatitis and hyperglycemia for 12.7% each. One-third of the toxicities were severe and occurred primarily during induction and consolidation phases [10].

Zahra et al. reported remission rates of 83-95% in pediatric ALL patients receiving asparaginase therapy, but also noted adverse effects, including hypersensitivity and cytotoxicity to the liver and pancreas, in 2.5-16% of patients [13]. Christ et al. compared PEG-asparaginase to L-asparaginase in adult ALL patients undergoing pediatric-inspired treatment. Among 48 patients receiving PEG-asparaginase, 60% developed hepatotoxicity, 17% pancreatitis, 19% thrombosis, and 71% experienced grade 3-4 toxicities. In contrast, the L-asparaginase group showed 33% hepatotoxicity, 22% pancreatitis, 0% thrombosis, and 44% grade 3-4 toxicities. The study concluded no statistically significant difference in grade 3-4 toxicities between the two treatment arms [14].

Our study is the first of its kind in this specific demographic and geographic context, assessing the incidence and risk factors for asparaginase-induced AP. A recent study published during our research addressed the broader adverse effects of chemotherapeutic agents in pediatric ALL. Our moderately sized cohort allows for robust and less biased conclusions. However, one limitation is our age cutoff of 14 years; including adolescents and young adults may have strengthened our findings.

Since AP incidence in our cohort commonly followed the third dose of asparaginase, we recommend standardizing pancreatic enzyme and triglyceride measurements prior to each dose. This could enable earlier detection and intervention, potentially preventing severe complications. Future prospective research should focus on monitoring pancreatic enzyme levels and lipid profiles to better understand the relationship between hypertriglyceridemia and asparaginase-induced AP.

## Conclusions

The incidence of AP occurring in children being treated for ALL in Saudi Arabia resembles the international data. Risk is elevated in older children, those on high-risk protocols, and those with multiple PEG-ASPA exposures. Early detection via monitoring and timely management can reduce morbidity. Future studies should investigate predictive biomarkers, genetic predisposing factors, and prevention strategies.
